# Selection of drug resistant mutants from random library of *Plasmodium falciparum *dihydrofolate reductase in *Plasmodium berghei *model

**DOI:** 10.1186/1475-2875-10-119

**Published:** 2011-05-10

**Authors:** Wachiraporn Tipsuwan, Somdet Srichairatanakool, Sumalee Kamchonwongpaisan, Yongyuth Yuthavong, Chairat Uthaipibull

**Affiliations:** 1 National Center for Genetic Engineering and Biotechnology (BIOTEC), National Science and Technology Development Agency (NSTDA), 113 Thailand Science Park, Pathumthani 12120, Thailand; 2 Department of Biochemistry, Faculty of Medicine, Chiang Mai University, Chiang Mai 50200, Thailand

## Abstract

**Background:**

The prevalence of drug resistance amongst the human malaria *Plasmodium *species has most commonly been associated with genomic mutation within the parasites. This phenomenon necessitates evolutionary predictive studies of possible resistance mutations, which may occur when a new drug is introduced. Therefore, identification of possible new *Plasmodium falciparum *dihydrofolate reductase (*Pf*DHFR) mutants that confer resistance to antifolate drugs is essential in the process of antifolate anti-malarial drug development.

**Methods:**

A system to identify mutations in *Pfdhfr *gene that confer antifolate drug resistance using an animal *Plasmodium *parasite model was developed. By using error-prone PCR and *Plasmodium *transfection technologies, libraries of *Pfdhfr *mutant were generated and then episomally transfected to *Plasmodium berghei *parasites, from which pyrimethamine-resistant *Pf*DHFR mutants were selected.

**Results:**

The principal mutation found from this experiment was S108N, coincident with the first pyrimethamine-resistance mutation isolated from the field. A transgenic *P. berghei*, in which endogenous *Pbdhfr *allele was replaced with the mutant *Pfdhfr^S108N^*, was generated and confirmed to have normal growth rate comparing to parental non-transgenic parasite and also confer resistance to pyrimethamine.

**Conclusion:**

This study demonstrated the power of the transgenic *P. berghei *system to predict drug-resistant *Pfdhfr *mutations in an *in vivo *parasite/host setting. The system could be utilized for identification of possible novel drug-resistant mutants that could arise against new antifolate compounds and for prediction the evolution of resistance mutations.

## Background

*Plasmodium falciparum*, the most virulent malaria pathogen species, is responsible for nearly 863,000 deaths in 2009 [1]. Malaria treatment is hampered by existence of only a limited number of drugs and the emergence of parasites resistant to most available antimalarial drugs, including chloroquine, pyrimethamine and proguanil. Therefore, there is an urgent need to search for affordable, effective and safe anti-malarials that can combat drug resistant parasites.

A bifunctional enzyme in the folate biosynthesis pathway, dihydrofolate reductase-thymidylate synthase (DHFR-TS) is a well-defined target of traditional antimalarial drugs such as pyrimethamine and cycloguanil [2]. DHFR catalyses the production of tetrahydrofolate from dihydrofolate while TS is in charge of transferring a methyl-group from N5, N10-methylene-tetrahydrofolate to dUMP thereby generating dTMP and tetrahydrofolate. Mutations in *Pfdhfr *gene, associated with the amino acid substitution at residues 51, 59, 108, and 164, have been found in the field with different levels of resistance to antifolate drugs [3,4]. These mutations are positioned around the enzyme active site. Thus, the accumulation of point mutations in *Pfdhfr *reduces the affinity of antifolate drugs such as pyrimethamine for the enzyme leading to drug resistance [5,6]. These correlations of drug resistance with mutations in *Pfdhfr *gene have been experimentally verified by comparison of mutant enzymes in transfected parasites [7]. Despite the emergence of resistant *Pfdhfr *mutants in malaria endemic areas, the elucidation and development of target based screening models [8,9] and the solution of the crystal structure of *Pf*DHFR-TS [10] means that this enzyme is still an attractive target for drug development. Therefore, in order to develop new compounds against drug resistant parasites, prediction of possible future resistance mutations becomes a priority.

Studies of mutant *Pf*DHFR-TS expression in non-*Plasmodium *surrogate systems, such as *Escherichia coli *and *Saccharomyces cerevisiae *have successfully identified novel mutant *Pfdhfr *alleles that confer antifolate drug resistance [8,11]. These systems have also been useful in testing new compounds against the enzyme [8,9]. However, the biochemical activities of some mutant enzymes isolated using these systems have been shown to be significantly lower than the wild-type enzyme [11]. The physiology of these surrogate cells are markedly different from *Plasmodium *parasites, and it is not clear whether these resistant enzymes would have sufficient enzymatic activity to support parasite growth throughout all stages of the life cycle. Unfortunately owing to the narrow primate host range, suitable *Plasmodium in vivo *models are not available.

Rodent malaria parasites such as *Plasmodium berghei *are attractive models for human malaria, and the recent advances in DNA transfection technology allow for genetic modification of the parasite [12,13]. DNA transfection can be used to express *P. falciparum *genes in *P. berghei*, and thus transgenic *P. berghei *parasites can be used as *in vivo *surrogate models. In this study, the power of the transgenic *P. berghei *system to predict drug-resistant *Pfdhfr *mutations in an *in vivo *parasite/host setting was demonstrated. The objective was to identify mutations in *Pfdhfr *that confer resistance to anti-folate drugs using a transgenic *P. berghei *model. Libraries of randomly mutated *Pfdhfr *were generated by PCR mutagenesis and transfected to *P. berghei*. Upon transfection, resistant parasites were obtained after pyrimethamine selection and found to harbor *Pfdhfr *mutant alleles. Thus, this system can be used as a *Plasmodium *surrogate system for more accurate prediction and identification of antifolate-resistance mutations.

## Methods

### Experimental animals and parasite

For all experiments, female BALB/c mice (National Laboratory Animal Center, Mahidol University, Thailand) 4-6 weeks old and weighing 20-25 g were used for *P. berghei *parasite infections. The transgenic *P. berghei *parasite line MRA-867 expressing green fluorescent protein without drug-resistant selectable marker (PbGFP), kindly provided by Drs. Andrew Waters and Chris Janse of Malaria Research Group, Leiden University Medical Center, the Netherlands, was used in this study [14]. All animal work was evaluated and approved by the Ethical Committee on Animal Experimentation, National Center for Genetic Engineering and Biotechnology (BIOTEC), Thailand, and followed international guidelines for the use of animals in experimental studies.

### Construction of *P. berghei *transfection plasmid

Plasmid for *P. berghei *transfection in this study was modified from the original plasmid pL0017 [15], which was kindly provided by Drs. Andrew Waters and Chris Janse (Leiden University Medical Centre, the Netherlands). The final transfection plasmid, designated pY005, contains wild type *Pfdhfr-ts *gene flanked with 1.0-kb each of 5' and 3' untranslated region (UTR) sequences of *Pbdhfr-ts. Bam*HI and *Afl*II restriction sites were introduced at 5' and 3' ends, respectively, of *Pfdhfr *domain to serve as cloning sites for the randomly-mutated *Pfdhfr *library.

### Construction of *Pfdhfr *random mutant library

*Pfdhfr *mutant library was generated by error prone PCR [11]. The 50 μL PCR reaction contained 1 ng of pY005 plasmid template harboring wild type *Pfdhfr*, 10 μM of sense primer F1 (CGGTGGATCCATGATGGAACAAG; *Bam*HI site is underlined), 10 μM of antisense primer R1 (CTTTGTCATCATTCTTAAGAGGC; *Afl*II site is underlined), 0.1 mM dGTP, 0.1 mM dATP, 0.5 mM dCTP, 0.5 mM dTTP, 1× Mutagenesis buffer [16] and 5 units of GoTaq^® ^DNA polymerase (Promega). The thermocycle condition was: 1 cycle of 95°C for 3 min, 30 cycles of 95°C for 1 min, 50°C for 1 min, 72°C for 1 min, and final extension of 72°C for 5 min. PCR products of random mutant *Pfdhfr *library of about 0.7 kb were cloned into the *Bam*HI/*Afl*II sites of pY005. The plasmids containing *Pfdhfr *mutation libraries were grown in Luria Bertani broth containing 100 μg/ml ampicillin in a 37°C incubator shaker for 12-16 hours. Plasmids were extracted and purified using a Qiaprep Spin Miniprep kit (Qiagen). Extracted plasmids were precipitated by isopropanol and re-suspended in 10 μl TE buffer (10 mM Tris, 1 mM EDTA pH 8.0) for use in transfection experiment.

### Determination of pyrimethamine sensitivity to parental and transgenic parasites

The sensitivity of pyrimethamine to inhibit the parental PbGFP parasite or transgenic parasites expressing *Pf*DHFR mutant was determined by the 4-day suppressive test [17]. Five groups of five BALB/c mice per group were infected intravenously (i.v.) with 1 × 10^7 ^parasitized erythrocytes and treated with different concentration of pyrimethamine by intraperitoneal (i.p.) injection four hours post infection. The control group was treated with 5% (v/v) DMSO in PBS pH 4.0. The experimental groups were treated with different doses of pyrimethamine through the same route for another 3 days. Twenty-four hours after the last treatment (day 4), percentages of parasitaemia were determined by microscopic counting of Giemsa-stained smears from mouse tail blood.

### Statistical analysis

All statistical analyses were carried out using SigmaPlot software version 11 (Systat Software Inc., USA). For calculation of the growth inhibitory curve, parasitaemia of the control group was set as 0% inhibition. The non-linear regression for sigmoidal dose-response (variable slope) was used to calculate the 50%, 90% or 95% effective dose (ED_50_, ED_90 _or ED_95_) values.

### Transfection, selection and identification of *Pfdhfr *random mutant libraries

*In vitro *culture of PbGFP and *P. berghei *transfection were performed as described [13]. Briefly, parasitized blood was collected from a donor animal and cultured overnight in culture media (RPMI 1640 medium containing 20% heat inactivated fetal calf serum, 50 IU/mL neomycin and 25 mM Hepes). Schizont stage parasites were purified from the overnight culture by Nycodenz gradient centrifugation. The merozoites were transfected with the circular plasmid DNA harboring *Pfdhfr *mutant libraries using the standard Amaxa Nucleofector protocol [13] and re-infected into animals by i.v. injection. Twenty-four hours after transfection, 0.25 mg/kg of pyrimethamine was used to treat the infected mice by i.p. injection daily. Smears were taken to check parasitaemia and when positive, left to multiply, until parasite numbers were adequate (about 3% parasitaemia) for genomic analysis. Tail blood was drawn from infected animals on alternate days until parasitaemia reached 8-10% and genomic DNA was extracted from whole blood using a genomic DNA Mini Kit (Geneaid). The genomic DNA obtained was transformed into *E. coli *DH5α to recover the circular plasmid DNA containing *Pfdhfr *mutants. The transformed bacterial colonies were picked and extracted for plasmid DNA using a Qiaprep Spin Miniprep kit (Qiagen). The sequences of *Pfdhfr *mutants were obtained by DNA sequencing (Biodesign Sequencing Service, Thailand).

### Transfection, selection and cloning of transgenic *P. berghei *parasite

Plasmid containing resistant mutant *Pfdhfr^S108N ^*was completely digested with *Hind*III and *Kas*I restriction enzymes to generate linear plasmid. The 5' and 3'UTRs of *Pbdhfr-ts *served as homologous recombination sites for replacement of the endogenous *Pbdhfr-ts *on chromosome 7 with *Pfdhfr^S108N^. In vitro *culture of PbGFP and *P. berghei *transfection were performed as described [13]. Twenty-four hours after transfection, 0.25 mg/kg of pyrimethamine was used to treat the infected mice by i.p. injection daily until the drug resistant parasites appeared. The integrated transgenic mutant parasite clones were obtained by the limiting dilution method [18].

### PCR analysis of transgenic *P. berghei *parasite

The correct integration of *Pfdhfr^S108N ^*sequence into the genome via the 5' and 3'UTRs of the *Pbdhfr-ts *locus was determined by PCR. A 4.0-kb DNA fragment spanning the endogenous 5'UTR *Pbdhfr-ts *sequence and the introduced *Pfdhfr *was indicative of an integration event and was amplified using primers F2 (TTGAGCTACATAACTTCCATACAT) and R1 (described above). A 3.0-kb DNA fragment spanning the introduced *Pfdhfr *and the endogenous 3'UTR *Pbdhfr-ts *sequence, indicative of a 3' integration event was amplified using primers F1 (described above) and R2 (CGATCTACACCTCTTCAT).

### Expression profile of transgenic mutant parasite

The transgenic parasites expressed *Pf*DHFR*^S108N^*-TS under the control of 5' and 3'UTRs of *Pbdhfr-ts*. To determine whether this promoter efficiently drives *Pfdhfr^S108N^-ts *mRNA expression, reverse-transcription PCR (RT-PCR) was performed. Total RNA was isolated from blood stage transgenic *P. berghei *parasites using Trizol reagent (Invitrogen). cDNA was generated and used as template for amplification with gene specific primers for the *Pfdhfr *transgene using primers F1 and R1 (described above). Controls included specific primers for *Pbdhfr *gene: PbDTF (GGGGGGGGCATATGGAAGACTTATCTGAAACATTCG) and PbDTR (GGACTAGTGTACTTCCTCATTTGG) and *P. berghei alpha tubulin *gene: PbatubulinF (GCATGCTGGGAGCTATTTTG) and PbatubulinR (GCTGGTTCAAATGCTGAGTTTG). RT-PCR was performed using the same PCR condition as described above.

## Results

### Determination of pyrimethamine sensitivity in wild type PbGFP parasites

**Figure 1:**
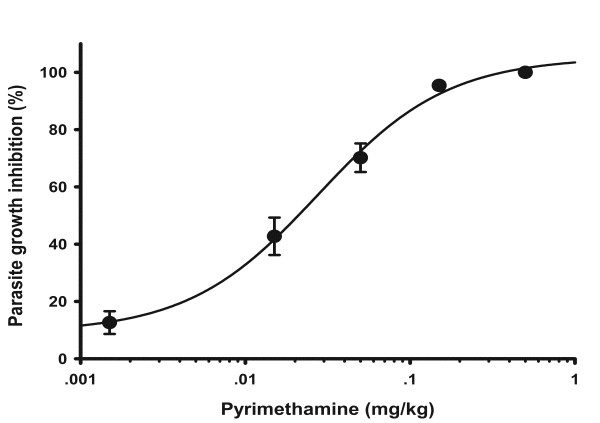
**Sensitivity of pyrimethamine against PbGFP parasite**. The data represents mean values ± SD of percentage of growth inhibition for 5 animals per group. The average ED_50 _from three independent studies is 0.02 mg/kg.

Experimental mice infected with wild type PbGFP parasites were treated with different concentrations of pyrimethamine. As shown in Figure 1, *in vivo *ED_50 _and ED_95 _of pyrimethamine against PbGFP was 0.02 mg/kg and 0.25 mg/kg, respectively. The ED_95 _concentration of pyrimethamine was then used for selection of transgenic resistant parasites in subsequent experiments.

### Construction of *Pfdhfr *random mutant library using wild type *Pfdhfr *template

Approximately 14,000 bacterial colonies were obtained for the *Pfdhfr *random mutant library. Twelve colonies were randomly picked and sequenced. From the sequence alignment, up to 4 base substitutions per gene were found. The mutation frequency was then calculated as 0.26%, which is equivalent to approximately 2 base substitutions per 700 bp of *Pfdhfr *gene. Thus, the PCR-induced mutagenesis for this library is within the expected mutation frequency of 2 to 5 base substitutions per gene [19]. The rest of the bacterial colonies were harvested for library DNA preparation.

### Selection of transfected resistant mutant parasites from wild type *Pfdhfr *random libraries

Plasmid DNA was purified from the *Pfdhfr *random mutant library and transfected to PbGFP parasites. Pyrimethamine-resistant parasites began to appear in most transfection experiments within 11 days post-transfection. Genomic DNA containing episomal transgenic DNA was extracted from drug-resistant parasites and transformed into *E. coli*. Among the isolated plasmids, two independent sequences were identified. One sequence contained a single base substitution mutation at amino acid position 108 (serine, S; AGC changed to asparagine, N; AAC) while the other had the same mutation at amino acid position 108 together with another base substitution at codon 196, which was silent (phenylalanine; TTT to TTC).

### Generation of transgenic *P. berghei *expressing *Pfdhfr^S108N^-ts *mutant

**Figure 2:**
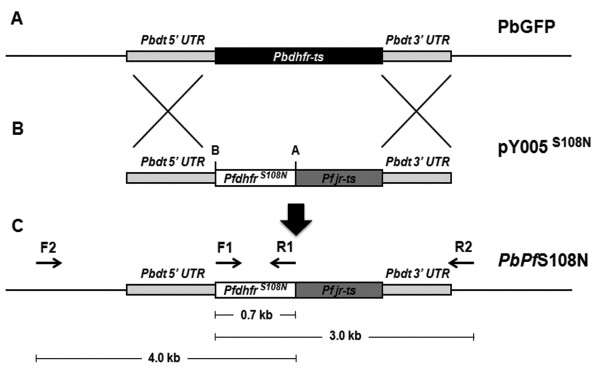
**Replacement strategy of mutant *Pfdhfr-ts *into *Pbdhfr-ts *by double cross-over homologous recombination**. (A) endogenous *Pbdhfr-ts *gene locus in PbGFP wild-type parasite, (B) linearized plasmid pY005 containing mutant *Pfdhfr^S108N^*, (C) *Pfdhfr^S108N^-ts *gene locus in transgenic *PbPf*S108N parasite. Positions of primers used for PCR amplification are indicated by arrows. The expected size of PCR products are described.

**Figure 3:**
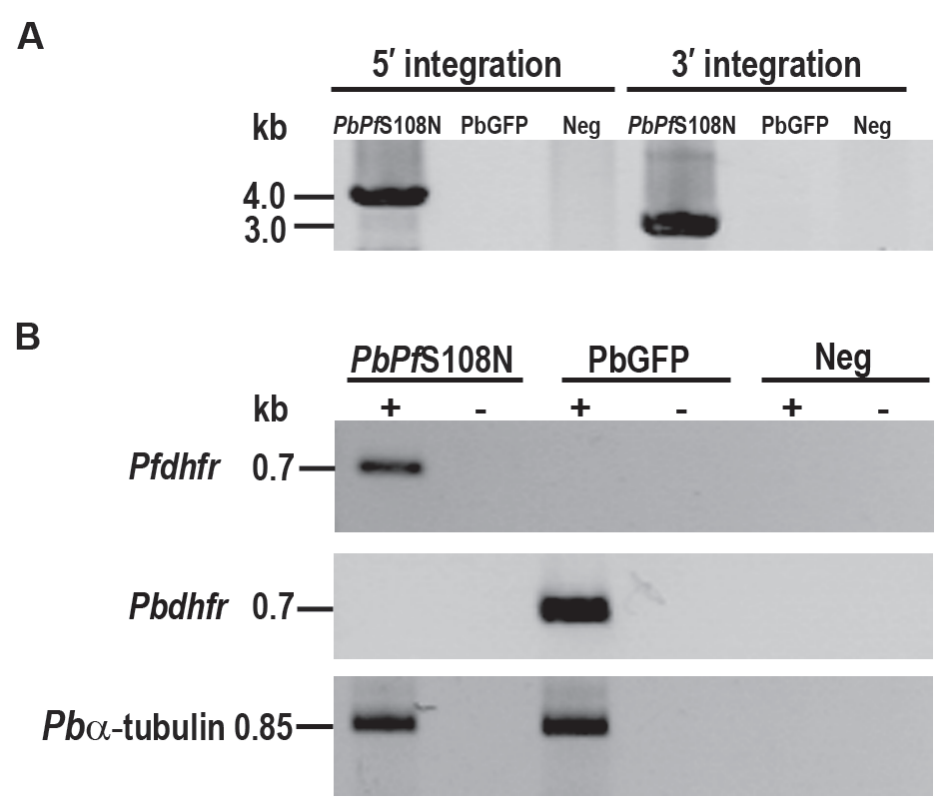
**PCR and RT-PCR analysis of the transgenic mutant parasite**. (A) PCR analysis of 5' and 3' integrations on genomic DNA isolated from transgenic mutant parasites, *PbPf*S108N, are shown in lanes 1 and 4, respectively. Genomic DNA isolated from PbGFP wild-type parasite and distilled water (Neg) served as negative controls as shown in lanes 2, 5 and 3, 6, respectively. (B) RT-PCR analysis of *PbPf*S108N parasites. RNA from the *PbPf*S108N parasite was reverse transcribed to cDNA and used as a template to amplify *Pfdhfr, Pbdhfr and P. berghei alpha tubulin *(*Pbα-tubulin*) genes. The reactions were performed with reverse transcription (lane 1), without reverse transcription (lane 2), *P. berghei *cDNA derived from PbGFP and distilled water (Neg) were used as negative controls with and without reverse transcription (lanes 3-6).

In order to confirm that the S108N mutation found among library-transfected DNA from drug-resistant parasites conferred resistance, allelic replacement of the endogenous *Pbdhfr-ts *with mutant *Pfdhfr^S108N^-ts *was performed. The strategy for the allelic replacement event is shown in Figure 2. Correct integration was investigated by PCR analysis on genomic DNA using different primers pairs. PCR products obtained corresponded with the expected 4.0 kb and 3.0 kb bands for 5' and 3'UTR integration respectively, as shown in Figure 3A.

### Expression profile analysis of transgenic *Pfdhfr^S108N ^*mutant parasite

Transgenic *P. berghei *parasite with integrated *Pfdhfr^S108N^-ts*, named *PbPf*S108N, expresses the gene under the control of *Pbdhfr-ts *5' and 3'UTRs. The expression of the enzyme was verified at the mRNA transcription level using RT-PCR. As shown in Figure 3B, 0.7 kb of *Pfdhfr *was amplified in cDNA from transgenic *PbPf*S108N parasite, but not in cDNA from PbGFP parental parasite. Conversely, *Pbdhfr *was amplified only in cDNA from parental PbGFP parasite, but not in cDNA from transgenic *PbPf*S108N parasites. The expected 0.85 kb band for the control *P. berghei alpha tubulin *was obtained for all parasites. No products were detected for -RT control templates, indicating that genomic DNA was absent.

### Determination of growth rate and pyrimethamine sensitivity in transgenic *PbPf*S108N parasite

**Figure 4:**
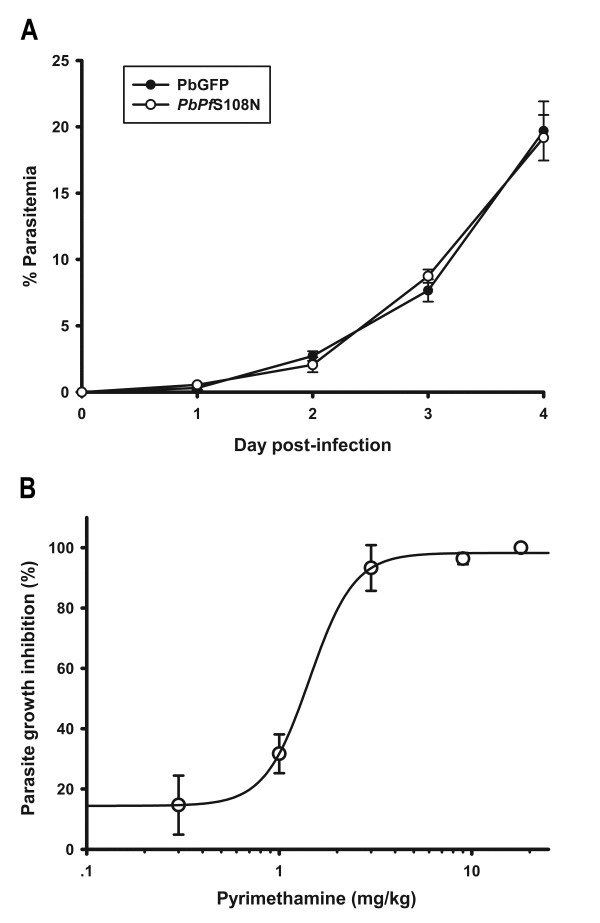
**Growth rate and pyrimethamine sensitivity of *PbPf*S108N parasite**. (A) Growth curves of *PbPf*S108N and PbGFP in mice. *PbPf*S108N data are represented as open circles and, PbGFP data represented as filled circles. The experiments were performed in three independent studies and the data represents mean ± SD values. (B) Sensitivity of pyrimethamine against *PbPf*S108N parasite. The data represents mean ± SD of percentage of growth inhibition. The average ED_50 _from three independent studies is 1.1 mg/kg.

The growth rate of transgenic *PbPf*S108N parasite was compared with PbGFP parental parasite. The growth rates of the two parasites were not significantly different (Figure 4A). The efficacy of pyrimethamine to inhibit transgenic *PbPf*S108N parasites was determined by 4-day suppressive test. The calculated ED_50 _and ED_90 _values of pyrimethamine against transgenic *PbPf*S108N parasites were 1.33 mg/kg and 2.65 mg/kg, respectively (Figure 4B).

## Discussion

There is need to identify possible drug-resistance mutations against new compounds under development as anti-malarial drugs. Prior knowledge of possible resistance pathways facilitates testing of strategies for forestalling the evolution and spread of resistance in the parasite population, e.g. drug combinations. In this study, a *P. berghei in vivo *model was developed as a surrogate cell expression system to identify *Pfdhfr *mutations that confer antifolate drug resistance. This system employs DNA transfection in which the selectable marker is antifolate-resistant mutant *Pfdhfr*, rather than the conventional *human dhfr *and *Toxoplasma gondii dhfr *markers used in *P. berghei *transfection [20]. The concentration of pyrimethamine at 0.25 mg/kg (ED_95 _of wild type *P. berghei *parasite) i.p. injection daily was successful in selecting resistant parasites obtained after transfection with *Pfdhfr *mutant libraries. Interestingly, the resistant parasite lines transfected with a *Pfdhfr *variant library were found to contain the S108N mutation, which is known to be the key antifolate resistance mutation found in nature [21]. The fact that the pyrimethamine resistant line carried this mutation provides proof of concept of this system to be able to identify drug resistant mutations from a mutant library.

*Pf*DHFR-TS crystal structure reveals the asparagine side chain of the mutant enzyme is in steric clash with the *p*-chlorophenyl moiety of pyrimethamine, which reduces the binding affinity of pyrimethamine for the mutant enzyme [10]. Although the selected drug-resistant library mutants contained the expressed S108N mutation, it should be noted that the drug-resistant library mutants are maintained episomally, and the increased *dhfr *gene copy number in episomes may contribute to drug resistance in transgenic *P. berghei*, as has been shown for the *hdhfr *transfection marker [20]. Thus, to test the hypothesis that pyrimethamine resistance in transgenic *P. berghei *is determined solely by the S108N mutant allele, *Pbdhfr-ts *was replaced with the *Pfdhfr ^S108N^-ts *mutant by double homologous recombination. The integrated transgenic parasites expressed *Pf*DHFR*^S108N ^*-TS with a single copy of mutant *Pfdhfr^S108N ^*under the control of endogenous *Pbdhfr-ts *5' and 3'UTRs. The resulting *PbPf*S108N transgenic parasites showed susceptibility to pyrimethamine with ED_50 _values 66-fold higher than PbGFP wild type, which strongly suggest that resistance is conferred by the S108N mutation. In addition, *PbPf*S108N transgenic parasites grew the same as PbGFP parasites. This result demonstrated that the DHFR-TS function is conserved between the two *Plasmodium *species, in agreement with other cross-species comparisons in the same genus [22]. Furthermore, the pyrimethamine resistance mutation had no negative effect on the function of the enzyme, in agreement with earlier studies [21,23]. This demonstrates the capacity of the transgenic *P. berghei *parasite model to study the fitness of *Pfdhfr *mutant alleles in the whole transmission cycle.

For pyrimethamine resistance, in addition to S108N mutation, other mutations such as N51I, C59R and I164L are known to augment the resistance level in parasites [21,23]. Although our mutant library is of sufficient diversity to contain these other mutations, they were not found among drug-selected parasites. In order to obtain transgenic parasites with a higher level of resistance, higher drug pressure will needed. However, selection of highly resistant pyrimethamine alleles with two or more mutations is not feasible, since mice could not tolerate the very high pyrimethamine doses needed.

In conclusion, a new experimental system for predicting the evolutionary pathway of antifolate drug resistance was developed. The major advantage of this system is that drug-resistant mutant alleles can be selected from diverse *Pfdhfr *libraries in a *Plasmodium *surrogate cell in a proper host background. Proof of concept for the system was demonstrated using the well-known antifolate pyrimethamine; however, new antifolates are being developed which are also effective against pyrimethamine-resistant parasites [24]. Therefore, the system could be utilized for identification of possible novel drug-resistant mutants that could arise against new antifolate compounds. This information could be used for rational design of effective anti-malarial drugs that forestall the emergence of drug resistance. Furthermore, our approach could also be applied to other *Plasmodium *enzyme drug targets for prediction the evolution of resistance mutations.

## Competing interests

The authors declare that they have no competing interests.

## Authors' contributions

CU, SS, SK and YY conceived and designed the project. WT performed all experiments. WT and CU prepared the manuscript. SS, SK and YY critically reviewed the manuscript. All authors read and approved the final manuscript.
